# Targeted Proteomics to Assess the Response to Anti-Angiogenic Treatment in Human Glioblastoma (GBM)*[Fn FN2]

**DOI:** 10.1074/mcp.M115.052423

**Published:** 2015-08-04

**Authors:** Kevin Demeure, Fred Fack, Elodie Duriez, Katja Tiemann, Amandine Bernard, Anna Golebiewska, Sébastien Bougnaud, Rolf Bjerkvig, Bruno Domon, Simone P. Niclou

**Affiliations:** From the ‡NorLux Neuro-Oncology Laboratory, Department of Oncology, Luxembourg Institute of Health, Luxembourg, Luxembourg;; §Genomics and Proteomics Research Unit, Department of Oncology, Luxembourg Institute of Health, Luxembourg, Luxembourg;; ¶KG Jebsen Brain Tumour Research Center, Department of Biomedicine, University of Bergen, Bergen, Norway

## Abstract

Glioblastoma (GBM) is a highly aggressive primary brain tumor with dismal outcome for affected patients. Because of the significant neo-angiogenesis exhibited by GBMs, anti-angiogenic therapies have been intensively evaluated during the past years. Recent clinical studies were however disappointing, although a subpopulation of patients may benefit from such treatment. We have previously shown that anti-angiogenic targeting in GBM increases hypoxia and leads to a metabolic adaptation toward glycolysis, suggesting that combination treatments also targeting the glycolytic phenotype may be effective in GBM patients. The aim of this study was to identify marker proteins that are altered by treatment and may serve as a short term readout of anti-angiogenic therapy. Ultimately such proteins could be tested as markers of efficacy able to identify patient subpopulations responsive to the treatment. We applied a proteomics approach based on selected reaction monitoring (SRM) to precisely quantify targeted protein candidates, selected from pathways related to metabolism, apoptosis and angiogenesis. The workflow was developed in the context of patient-derived intracranial GBM xenografts developed in rodents and ensured the specific identification of human tumor *versus* rodent stroma-derived proteins. Quality control experiments were applied to assess sample heterogeneity and reproducibility of SRM assays at different levels. The data demonstrate that tumor specific proteins can be precisely quantified within complex biological samples, reliably identifying small concentration differences induced by the treatment. In line with previous work, we identified decreased levels of TCA cycle enzymes, including isocitrate dehydrogenase, whereas malectin, calnexin, and lactate dehydrogenase A were augmented after treatment. We propose the most responsive proteins of our subset as potential novel biomarkers to assess treatment response after anti-angiogenic therapy that warrant future analysis in clinical GBM samples.

In the context of glioblastoma (GBM)^1^, the quest for effective biomarkers is vital given that GBM is the most aggressive primary brain tumor in adults and no curative treatment is currently available (1). GBM is characterized by extensive invasion into the brain parenchyma, a high proliferation rate, neo-angiogenesis and significant cellular and molecular heterogeneity. Current treatment involves neurosurgery, radiotherapy and chemotherapy, yet the median life expectancy of affected patients is less than fifteen months. Recent efforts have focused on targeting the vascular endothelial growth factor (VEGF) system which is critical for tumor angiogenesis, however GBM quickly develop escape mechanisms leading to tumor progression (2, 3). Previous work from our group demonstrated that GBMs adapt to anti-VEGF treatment via a metabolic switch in tumor cells toward increased glycolysis (4, 5). This was accompanied by increased hypoxia and tumor cell invasion, with little or no effect on tumor growth (4). In agreement with these preclinical studies, two large scale clinical trials addressing the impact of bevacizumab, a VEGF targeting antibody, in newly diagnosed GBM patients reported disappointing results: although progression free survival appeared to be improved, no effect on overall survival was observed (6, 7). The evaluation of such studies are complicated by the fact that anti-angiogenic agents affect blood vessel permeability thereby directly modulating neuroimaging parameters used to determine treatment effects (8, 9). Thus there is a need for molecular biomarkers to adequately determine treatment response to anti-angiogenic agents.

MS-based proteomics (10, 11) is widely used in the field of cancer research in particular in the context of biomarker development including discovery and verification. The application of the selected reaction monitoring (SRM) approach to proteomics reinforced the importance of MS in biomarker development (12–14). Indeed, SRM is a targeted proteomics approach that allows a precise and absolute quantification of previously selected marker candidates (15, 16). Moreover it can be applied in a supervised discovery phase for potential biomarkers (17, 18), *i.e.* the precise quantification of a wider range of selected biomarkers of interest by the use of stable isotope labeled (SIL) peptides in crude quality. Because of its high selectivity, sensitivity and accuracy, SRM, also named multiple reaction monitoring (MRM), is currently the reference method in targeted proteomics (14, 19).

The aim of this study was to identify proteins that are altered by anti-angiogenic treatment, thereby providing biomolecular signatures of tumor response in GBM. Ultimately such protein markers could be evaluated for their utility as markers of efficacy that allow to discriminate responders from nonresponders. The study was focused on target proteins that may exhibit significant differences in protein expression reflecting the metabolic switch exhibited during anti-angiogenic therapy. An SRM workflow designed on a triple quadrupole platform (20), was developed and optimized in the context of GBM xenografts treated with bevacizumab in order to perform, in a supervised manner, a precise relative quantification of target proteins. We have previously shown that patient derived GBM xenografts developed in rodents faithfully reflect human pathology and allow a detailed analysis of the tumor and stromal compartments (4, 21–24). Furthermore xenograft models facilitate the access to control samples as well as the possibility of controlled interventional studies (25). The results presented herein demonstrate the feasibility of SRM to precisely quantify small changes in protein concentration after treatment. We highlight the importance of peptide selection, data normalization and consideration of the variability of target proteins within complex biological samples before assessing their concentration changes in subsequent comparative studies. From an initial set of 100 candidates, we screened 74 proteins and identified 32 responsive to anti-angiogenic treatment. We propose malectin, calnexin, lactate dehydrogenase A (LDHA), and isocitrate dehydrogenase (IDH) as novel response markers to anti-angiogenic therapy.

## EXPERIMENTAL PROCEDURES

### 

#### 

##### Patient Material

Glioblastoma samples were collected at the Neurosurgery Department of the Centre Hospitalier in Luxembourg (CHL, Luxembourg) or at the Haukeland University Hospital in Bergen (Norway) from patients having given their informed consent. Collection and use of patient tumor material was approved by the National Ethics Committee for Research (CNER) of Luxembourg, and by the regional ethical committee of the Haukeland University Hospital in Bergen, respectively.

##### Patient Derived GBM Xenografts and Anti-angiogenic Treatment

Patient derived GBM xenografts were generated in mice (21) or in rats (4, 5) as previously described. Briefly, organotypic tumor spheroids, derived from patients P3, P13, and T16, were cultured for 7–10 days until they reached a diameter of 200–300 μm. Fresh tumor spheroids were implanted into the right frontal cortex (5–6 spheroids/mouse in NOD/Scid immunodeficient mice; 8–10 spheroids/rat in Rowett nude rats (RNU). Tumor take was verified by MRI 3 weeks postimplantation and animals were stratified into control and treatment groups. Rats from the treatment group received weekly intravenous injections of bevacizumab (10 mg/kg, tail vein), whereas mice received weekly intraperitoneal injections of bevacizumab (20 mg/kg) for 3 weeks. Control animals received saline following the same schedule. At the end of the period, the animals were sacrificed, the brains were dissected out and cut into two pieces along the coronal plane at the tumor core. One piece was fixed for histology, the other was snap frozen in liquid nitrogen for protein analysis. The handling of the animals and the surgical procedures were performed in accordance with the European Directive on animal experimentation (2010/63/EU) and the Norwegian Animal Act and were approved by the national authorities responsible for animal experiments.

##### Tissue Lysis

Efficient tumor tissue disruption and homogenization was performed with magnetic beads (5 mm stainless steel) in a TissueLyser II tissue homogenizer (Qiagen). For each tissue sample, one magnetic bead was introduced into the Eppendorf tube followed by 20 μl of ice-cold buffer per mg of tissue (MS-friendly extraction buffer: Urea 8 m/Tris 30 mm (pH 7.5–8) supplemented with PhosStop (Roche) and protease inhibitor (Roche, EDTA free)). Tissue samples were immediately disrupted (TissueLyser II parameters: 2.5 min at 20Hz, twice if needed), the beads removed, the samples briefly vortexed and gently mixed in a Thermomixer (Eppendorf Belgium, Rotselaar, Belgium) at 4 °C during 45 min. To further improve sample homogenization, two freeze-thaw cycles (−80 °C/ice-cold) and an ice-water bath sonication step (Bioruptor (Diagenode Europe, Seraing, Belgium) at medium sonication intensity (ON/OFF pulse time of 30 s for 5 min) were performed. Samples were centrifuged at 15,000 rcf (12,700 rpm) during 15 min (4 °C) to remove cell debris and unsolubilized components. Supernatants were transferred to fresh Eppendorf tubes and stored at −80 °C prior further treatment.

##### Protein Extraction and Digestion

To get rid of lipids, detrimental to the LC-MS setup, proteins were precipitated using methanol/chloroform/water (26). After precipitation (150 μl of protein extract per sample), the pellet was air dried for several minutes (5 min maximum) and solubilized in 200 μl Urea 8 m/Tris 30 mm until complete solubilization in a Thermomixer (Eppendorf Belgium) at 25 °C (1000 rpm). Total protein concentrations of samples were estimated by 2D-Quant assay (Bio-Rad, Temse, Belgium) according to the manufacturer's instructions. 20 μg of total protein extract was diluted in Urea 8 m/Tris 30 mm to a final volume of 150 μl (pH 8.0–8.5). 14 μl of 150 mm DTT (dithiothreitol) (in 50 mm ABC (ammonium bicarbonate) (pH 7.5–8.0)) was added and samples were reduced for 30 min at 37 °C (800rpm, Thermomixer (Eppendorf Belgium)). Nineteen microliters of 400 mm IAA (iodoacetamide) (in 50 mm ABC (pH 7.5–8.0)) was added and samples were alkylated for 30 min at 37 °C in the dark (800 rpm, Thermomixer (Eppendorf Belgium)). Samples were subsequently diluted to 1 m Urea by adding 1018 μl of 50 mm ABC (pH 7.5–8) and pH was adjusted to 7.0–8.0. Two micrograms (1:10 (w:w) (enzyme:protein)) of sequencing grade modified porcin trypsin (Promega, Charbonnieres, France) was added and samples were digested overnight at 37 °C (1000rpm, Thermomixer (Eppendorf Belgium)). Digestion was quenched by acidifying samples at a pH of 2–3 with 10% formic acid. Protein digests were cleaned up with Sep-Pak tC18 SPE cartridge (Waters, Guyancourt, France) according to the manufacturer's instructions and were concentrated using a SpeedVac (Thermo Scientific) and kept lyophilized until analysis.

##### LC-MS/MS

Lyophilized samples were solubilized in 40 μl formic acid 0.1% (in HPLC grade water) for the SRM screening experiments (see Peptide Selection section for further information regarding the selection of the proteotypic peptides) or in 40 μl of the pool of stable-isotope labeled (SIL) peptides (formic acid 0.1%; [Peptide Retention Time Calibration Mixture (PTK15) (Thermo Fisher)] = 15 fmol/μl) for the precise relative quantification experiments of untreated and treated xenograft samples. Peptides were separated in a dual nanoflow liquid chromatography system (two trap columns and two analytical columns allowing high-throughput analyses) coupled to the electrospray source of a triple quadrupole platform, TSQ Vantage extended mass range (Thermo Scientific, San Jose, CA), to perform SRM experiments. One microliter of each samples was first loaded onto a trap column (Acclaim PepMap 2 cm × 75 μm ID, C18, 3 μm, 100 Å (Dionex)) at 5 μl/min with aqueous 1% acetonitrile and 0.05% trifluoroacetic acid (TFA) for 3 min. Subsequently, the trap column was set online with the analytical column and trapped peptides were eluted on the analytical column (Acclaim PepMap RSLC 15 cm × 75 μm ID, C18, 2 μm, 100 Å; Dionex). The 53 min gradient was divided as 2% solvent B (HPLC grade acetonitrile (0.1% (v/v) formic acid)/98% solvent A (HPLC grade water (0.1% (v/v) formic acid) over 10 min for equilibration, solvent B proportion was ramped linearly from 2% to 35% over 48 min for peptides separation at a flow-rate of 300 nL/min and followed by 90% solvent B over 5 min for washing. The dual-LC system avoids the waiting periods during column equilibration between two subsequent injections because the equilibration step was performed in parallel on the unused channel (column washed by 90% solvent B during the gradient on the other channel). Samples were analyzed in triplicates and each replicate consisted of four injections (two injections on each channel) to monitor all the transitions. Each replicate analysis was separated by the analysis of a QC sample (PTK15 diluted at 25 fmol/μl in HPLC grade water) on each channel for quality control of instrument performance.

##### Peptide Selection for the Targeted Protein Candidates

For this supervised study, a list of 100 protein candidates was established based on previous intralaboratory data (transcriptomics and prior proteomics experiments for protein identification by shotgun proteomics on GBM xenografts) and literature mining. This also included proteins that we have previously shown to discriminate angiogenic tumors from invasive tumors (24), as shown in supplemental Table S1. Signature peptides, surrogates of the targeted proteins, were selected for the set of 100 proteins. Because of the sample origin (human tumor transplanted within the brain of a mouse or a rat) the choice was further restricted to signature peptides that were human specific and whose sequence was not present in the rodent proteome. However, in some cases, when only very few peptides (1–2 peptides) suited these criteria, the selection was partly broadened and signature peptides that were present in either rat or mouse proteome were added. To facilitate and expedite this selection step, an *in-house* software, named *PeptideManager*, combining data from two protein databases was developed (27). This software provides the peptide sequences and available related information of a given protein in a proteome of interest as well as the occurrence for each peptide sequences within a host/background proteome. The human proteome (UniProt Human database version: 07_2011) was used as proteome of interest and the rodent proteome (UniProt Mouse database version: 07_2011 and the UniProt Rat database version: 07_2011) as host/background proteomes. Peptide selection was performed following criteria previously reported (15) as well as the additional criteria of sequence uniqueness within the proteome of interest (human) and its absence in the host/background proteome (mouse, rat and/or both). For each protein, as many species-specific peptides as possible were selected. When a large number (typically more than five) of peptide candidates were available for a given protein, the MS-behavior of these peptides were estimated with the help of PeptideAtlas (28) allowing to rationalize further peptide selection. The peptides with the best LC-MS behaviors should lead to MS measurements with a higher sensitivity. As far as possible, peptide candidates present at different locations within the protein sequence (*e.g.* first third, middle part, and last third) were selected. Finally, peptide selection was based on our own experimental evidence via preliminary SRM screening experiments on a triple quadrupole platform. This screening consisted in monitoring as many transitions as possible for each selected endogenous peptide through the whole LC gradient. Only screened peptides, which gave co-eluted signals (for the different transitions) of significant intensities, were kept.

##### SRM Assay Development

For the selected peptides, the corresponding SIL peptides were synthesized in crude quality (Thermo Fischer). SIL peptides were analyzed in water in order to select the four most intense transitions for each peptide, to determine their response factor and the retention time at which they were observed. Based on this, SIL peptides were pooled at concentrations of 50 nmol/μl or 200 nmol/μl. The retention times were used to establish time windows of 4.5 min around each peptide in order to design scheduled SRM assays that permit to monitor more transitions per LC-run. The internal standards (SIL peptides) were used to confirm the detectability of each endogenous peptide within the samples. The correspondence between the endogenous peptide and its corresponding SIL peptide of their retention times and their fragmentation patterns (relative intensities of the different transitions monitored) were used as validation conditions.

##### Data Analysis

The data extraction (from raw files), the peak picking and the data normalization (with the SIL peptides) were automated thanks to our *in house* developed software (*SRManager*). Briefly, a database containing all the extracted SRM traces for an entire experiment with all the information needed and related to it (the SRM assay experimental design) was built by SRManager. With the help of the peptide trainer kit (PTK15) spiked in each sample analyzed, the retention times of all the SRM traces were realigned on “standardized” retention times (retention times determined in a QC sample acquired in between the analysis of the biological samples) of the 15 peptides of the PTK15 calibration mixture. The peak picking was based on the “multiplied trace” created by multiplying together all the traces from a given peptide. For each SIL peptide, the multiplied trace was used to determine up to three best maxima (with scores). Within a small time window around the maxima of the signal (the multiplication of its SRM traces) of a SIL peptide, the traces of the corresponding endogenous peptide was scrutinized to determine the presence of co-eluting signals which are then scored. The peak selection was based on the co-elution of the traces of the SIL peptides and of the endogenous peptides, the S/N of the selected peaks and the dot product (with the area of the selected peaks) between the SIL transitions and the endogenous transitions.

The normalization procedure of the data was based on the SIL peptides and consisted in two steps. For each sample and for each raw file (because the complete assay was composed of 4 LC-runs (4 different raw files; raw files #1, #2, #3, and #4)), the median of the log_2_ transformed areas of all the transitions of the SIL peptides were calculated after having removed the outliers (determined using the lower and upper inner fences). For the 4 different raw files, an overall median was calculated using the median of each samples. Those four overall medians were used to calculate the first normalization factors (overall median [raw file #i]/median [raw file #i] in sample x) to apply to the areas of the transitions of the SIL and endogenous peptides. After this first normalization step, the overall median of the areas of all the transitions of a SIL peptide within all the samples were calculated for each SIL peptides after having removed the outliers (determined using the lower and upper inner fences). Those overall medians were then used to calculate the second normalization factors for each peptide in each sample (overall median [peptidei]/median [peptide i] in sample x) to apply to the areas of the transitions of the SIL and endogenous peptides. For both normalization steps, if the normalization factor exceeded a 2.5 factor, the data point was considered as an outlier and discarded.

The normalized dataset was then used to evaluate the consistence of the transitions of the endogenous peptides through all the samples via the use of the Cronbach's alpha, as developed by Duriez *et al.* (manuscript in preparation) as well as their correlation via the use of Spearmann and Pearson. Transitions with Cronbach's alpha superior to 0.9 were conserved as well as transitions with Cronbach's alpha between 0.8 and 0.9 having a Spearmann factor superior or equal to 0.7 and a Pearson factor superior or equal to 0.75.

The data was further normalized with supposed (in the context of this study) human house-keeping proteins (SwissProt accession numbers: P04406 (Glyceraldehyde-3-phosphate dehydrogenase (GAPDH)), P18124 (60S ribosomal protein L7 (RPL7)), P47914 (60S ribosomal protein L29 (RPL29)) Q02878 (60S ribosomal protein L6 (RPL6)) in order to take into account the variable proportion of human and mouse/rat protein content within the different samples. Normalization factors of a sample were calculated by dividing the area of a transition in this sample by the mean area of this transition among all the samples, and this, for each transition of each endogenous peptide related to the house-keeping proteins. For each sample, the mean of these normalization factors was used to normalize the data. This correction was performed by multiplying all the measured transition areas by the corresponding normalization factor (those normalization factors are technical replicate-specific).

Concerning the relative quantification between treated and untreated samples, the comparison was done at the transition level. Therefore, each peptide can give up to fourfold change values if all its transitions were conserved. For each fold change value, *p* values were estimated with student *t* test when normality (Shapiro-Wilk) and equal variance tests were passed and, with Mann-Whitney Rank Sum Test when one of both tests failed. Multiple comparison correction was performed with the Bonferroni procedure.

##### Western Blots

Protein extracts from rat xenograft tissue were resolved on 4–12% BisTris gel (NP0323, Lifetech, Gent, Belgium) and transferred on PVDF membranes (Invitrolon PVDF, Lifetech, # LC2005). Blocking of the membrane was done with 2% milk in Tris-buffered saline solution with 0.1% triton X-100. Protein detection was done with the following primary antibodies: Anti-malectin C-terminal (1/6000, SIGMA, Diegem, Belgium, # SAB4200245) and anti-IDH1 (1/1000, DiaNova, Hamburg, Germany, # DIA-W09). Normalization of the signal were done with actin (detection with anti-actin, clone C4 (1/10000, millipore, # MAB1501)). Secondary antibodies (peroxidase-conjugated) were respectively: anti-Rabbit IgG (H&L) (Jackson ImmunoResearch, Suffolk, UK, #111–036-003), anti-Rat-IgG (H+L) (DiaNova, # *112–035-062*), ECL Anti Mouse IgG (GE healthcare #NA931V/AG). Signal detection was performed with Super Signal West Femto Maximum Sensitivity Substrate-(Thermo SC # 34095) according to manufacturer's instructions. Image acquisition was done with an ImageQuant LAS4010 imaging station and signal intensities were quantified with Image Quant TL software (GE Healthcare, Belgium).

## RESULTS

### 

#### 

##### Target Proteins and Surrogate Peptide Selection

SRM assays were designed on a triple quadrupole platform in order to perform, in a supervised manner, a precise relative quantification of targeted proteins in human GBM after bevacizumab treatment. A complete SRM workflow is provided in the context of intracranial GBM xenografts developed in rodents, taking into account the presence of mixed proteomes in the experimental design (*i.e.* the selection of the surrogate peptides and the normalization procedure of the data). The selection step of surrogate peptides was expedited and facilitated by the use of *PeptideManager*, a software developed in our laboratory (27). *PeptideManager* provides the peptide sequences and available related information of a given protein in a proteome of interest, as well as the occurrence for each peptide sequence within a host/background proteome (mouse or rat such as in the case of xenografts). Thus the present setup ensures that the proteins determined are guaranteed to be tumor derived and do not emanate from the stroma. The starting list consisted of 100 protein candidates including relevant candidates for the discrimination of angiogenic and invasive tumors (4, 24), from previous transcriptomic data (29) and preliminary proteomics data (protein identification by shotgun proteomics on GBM xenografts). Ten proteins were discarded because no tryptic peptides were found unique to the human proteome and absent from the rodent proteome as assessed by *PeptideManager*. For the remaining 90 proteins (supplemental Table S1), 587 surrogate peptides were selected and preliminary screening SRM experiments were performed to evaluate peptide detectability. Based on experimental evidence, 269 peptides (surrogates of 74 proteins) exhibited co-eluted traces of significant intensities and were maintained for the next step. The corresponding SIL peptides were used to confirm that the co-eluted signals corresponded to the endogenous peptide of interest. The selected peptides are indicated in supplemental Table S1. Moreover, the monitoring of the SIL peptides was used to determine retention time windows of 4.5 min around the signal of interest for the multiplexing of the SRM assay. For the 269 endogenous peptides and their corresponding SIL peptides, 2152 transitions were monitored (four transitions for the endogenous peptide and four transitions for the corresponding SIL peptide) distributed within four SRM methods (*i.e.* four injections of the sample, four MS analysis, four raw files) in order to keep a sufficient data sampling rate. The list of the transitions monitored in the SRM assay can be found in supplemental Table S2. The thus developed and optimized SRM assay was subsequently applied for the precise relative quantification of the 74 proteins in control and bevacizumab treated GBM xenografts.

##### Assessing Variability of Target Proteins within Biological Samples

**Fig. 1.**
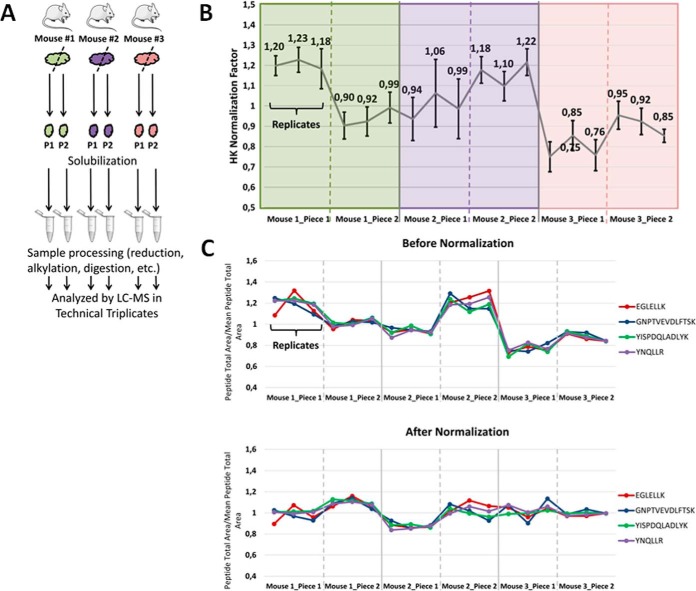
**Experimental design and assessment of normalization factors in xenograft samples.** *A*, Experimental design to assess the heterogeneity of GBM xenografts in mice. Three untreated GBM xenografts (same lot of tumor spheroids) with two tumor pieces each were used for protein heterogeneity analysis. Each sample was analyzed in technical triplicates in the designed SRM assay. *B*, Four human house-keeping (HK) proteins (SwissProt accession numbers: P04406, P18124, P47914, Q02878) were monitored in order to normalize the variable mouse/human protein amount within each sample. The normalization factors were obtained by dividing the areas of the transitions of the peptides, surrogates of the house-keeping proteins, within one sample replicate by the average areas of these transitions among all the samples. The diagram shows the average house-keeping normalization factors estimated for each sample replicate. The CVs of these normalization factors do not exceed 15%. *C*, Effect of the house-keeping protein normalization procedure on the quantification of alpha-enolase (P06733). For a given peptide within a given sample replicate, the ratio to the average was calculated by dividing the summed areas of the transitions of the peptide in the given sample replicate by the average of these summed areas among all the sample replicates. Those ratios for the different peptides monitored for alpha-enolase (14 transitions in total corresponding to four peptides) are indicated for the different samples before the normalization procedure (upper diagram) and after the normalization procedure (lower diagram). The intra mouse variability ranges from 7 to 20% before and from 1.2 to 10.2% after normalization. The inter-mouse variability, ranging from 15.5 to 20.4% before the normalization, is reduced to 7–11.1% after normalization. This normalization procedure is intended to take into account the variable proportion of mouse/human protein content within each sample. Therefore, the estimated variability of the proteins after the normalization procedure should represent their variability within the human protein content of the samples.

**Fig. 2.**
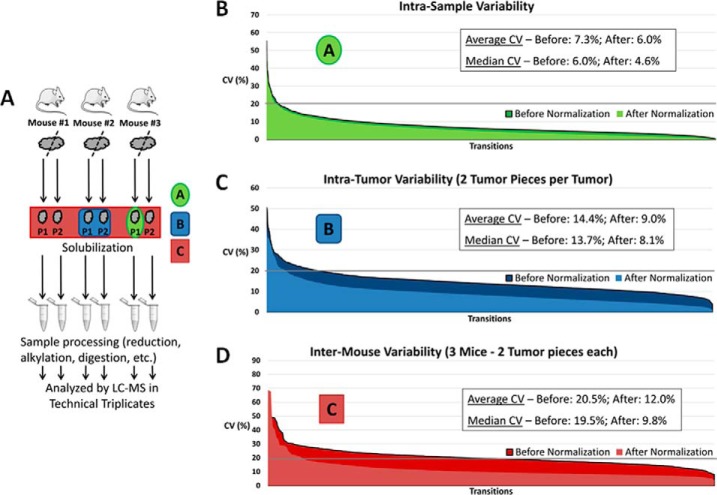
**Heterogeneity assessment of the proteins of interest within GBM xenografts.** *A*, Experimental set up indicating the different levels of heterogeneity analyzed, as indicated by different colors (green: intrasample, blue: intratumor, red: intermouse variability). *B*, Intrasample variability (technical replicates): the CVs of each transition for every endogenous peptide within each tumor piece for all samples are indicated in decreasing order before and after normalization (2754 transitions/measures in total). *C*, Intratumor variability: the CVs of each transition for every endogenous peptide within geographically distinct tumor pieces of each mouse are indicated in decreasing order before and after normalization (1377 transitions/measures in total). *D*, Intermouse variability: the CVs of each transition for every endogenous peptide within all the mice are indicated in decreasing order before and after normalization (459 transitions/measures in total). The values of the average and median CVs before and after normalization are indicated within each diagram. The effect of the normalization is minor at the intrasample level (diagram *A*) but far more significant at the intratumor and intermouse levels (diagrams *B* and *C* respectively).

Because of the high heterogeneity of biological samples, in particular human cancer tissue, it is crucial to estimate the variability of a targeted protein within a given sample before performing relative quantification experiments. The variability of the proteins of interest was assessed on tumor pieces derived from three different mouse xenografts generated from the same human GBM (Fig. 1*A*). Three levels of sample variability were assessed: (1) between the technical replicates of a same tumor piece (intra sample variability), (2) between regionally different tumor pieces derived from the same xenograft (intratumor variability), and (3) between tumors derived from separate mice (inter-mouse variability) (Figs. 1*A* and 2). Because the relative protein amounts derived from human tumor and mouse stroma can differ in each sample, normalization factors per sample replicate were calculated for each transition for all the endogenous peptides related to the human house-keeping proteins as shown in Fig. 1*B*.

To determine the impact of this normalization, the different levels of variability were assessed with and without correcting for the human/host protein content. All normalization factors, based on the validated transitions of the surrogate peptides of the house-keeping proteins (see data analysis section), were well correlated and their means were used for the normalization procedure. The CVs of the normalization factors used did not exceed 16% (supplemental Table S3). An illustration of the normalization effect is shown for alpha-enolase (P06733 protein) monitored by four peptides (14 transitions) (Fig. 1*C*). Before the normalization procedure, at the transition level, the CVs at the intermouse level were between 15.5% and 20.4%. After the normalization, the CVs were comprised between 7% and 11.1%. At the intratumor level (1 mouse, 2 tumor pieces), the median CV is reduced from 10% to 7.1%. The normalization procedure also decreased slightly the variability at the intrasample level (3 technical replicates by tumor piece) from a median CV of 4% to a median CV of 2.7%. The aim of this normalization step was not to obtain a maximal decrease in CV value, but to obtain corrected CV values that were representative of the variability of the target human protein within the xenograft independent of the variable proportion of human/mouse protein content for each sample.

In the global dataset, the CVs of all the transition measurements of all the endogenous peptides were estimated for the various samples at the different levels, as depicted in decreasing order in the graphs of Fig. 2. The median and average CVs were slightly reduced at the intrasample level (Fig. 2*B*) but were more significantly decreased at the intratumor (Fig. 2*C*) and at the intermouse (Fig. 2*D*) levels. For the vast majority of the endogenous peptides monitored (2693 transitions out of 2754 transitions in total), the transition measurements exhibited CVs below 20% at the sample level (Fig. 2*B*). The intermouse variability values were used to determine whether a fold change observed for a peptide between treated and untreated samples was higher than the biological variability of this peptide among mouse xenografts. The same graphs at the peptide level (*i.e.* the sum of the areas of the transition measurements of the peptide) are available in supplemental Fig. S1. The results of the variability assessment of the protein candidates within mouse xenograft samples are shown in supplemental Table S4 (at the transition level and at the protein level (average and median CVs)).

##### Relative Quantification of Target Proteins in GBM Xenografts After Bevacizumab Treatment

**Fig. 3.**
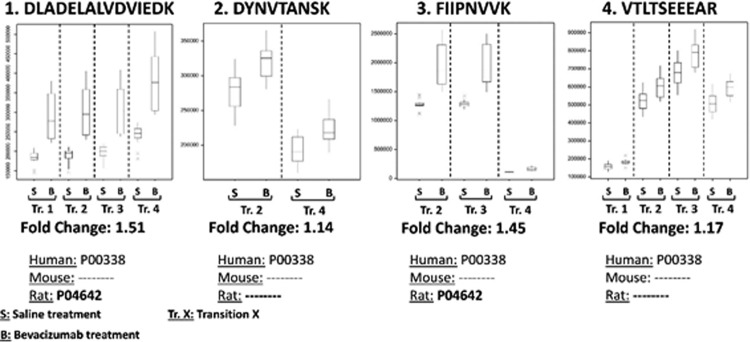
**Species-specific monitoring of lactate dehydrogenase A (LDHA) in rodent xenografts.** Boxplots of the different transitions monitored for the four peptides of human LDHA protein in rat xenografts with saline (S) or bevacizumab (B) treatment (4 samples in triplicate). Peptides 1 and 3 are present in the rat proteome whereas peptides 2 and 4 are specific to the human proteome. The different fold change values obtained for peptides 1 and 3 demonstrate the crucial importance of a cautious selection of the surrogate peptides in the context of samples with mixed proteomes such as in xenografts. Those fold change values indicate also that the rat LDHA is also increased in rat host tissue after bevacizumab treatment.

To determine the impact of bevacizumab in GBMs, SRM assays for the 74 proteins of interest were first applied to GBM xenografts generated in mice which were considered as test material (a total of six tumor pieces from four different mice per condition, saline or bevacizumab treated. This was followed by a validation step in GBM xenografts generated in rats (a total of four tumor pieces from four rats per condition). Both datasets were processed in the same manner as described under “Experimental Procedures.” They only differed by a few peptides that were detected in either rodent proteome in addition to the human proteome, where those peptides were only taken into account in the dataset where they accurately discriminated human from host. Fig. 3 illustrates the importance of this species-specific peptide selection. Four peptides of the human l-lactate dehydrogenase A chain (LDHA; P00338 protein) were monitored in mouse and rat xenografts. Although the overall trend indicating an up-regulation of LDHA after bevacizumab treatment, was identical for the four peptides, the fold change was much higher for peptides (1) and (3) (Fig. 3). Interestingly, although all four peptides were absent from the mouse proteome, peptides (1) DLADELALVDVIEDK and (3) FIIPNVVK were detected in the rat proteome, indicating that the value reported by these peptides represents a cumulative effect of the protein from human tumor and rat stroma. Therefore in the case of the rat xenograft dataset, only peptides (2) and (4) were considered. It is noteworthy that fold change values obtained with peptides (1) and (3) were very similar, which indirectly confirmed that the normalization procedure with human house-keeping proteins had successfully taken into account the different human/rat protein contents across samples.

At the biological level, it is important to note that the present approach demonstrates increased levels of LDHA not only in the tumor tissue but also in the stroma. We have previously reported an increase in LDHA in bevacizumab treated GBM correlating with increased lactate levels in the tumor (5). The current species-specific targeted proteomics approach confirms and expands on these data by incorporating the non-neoplastic tissue, suggesting that the glycolytic switch is not limited to tumor cells but encompasses the surrounding stroma *e.g.* neural tissue, endothelial cells, pericytes and/or microglia/macrophages.

**Fig. 4.**
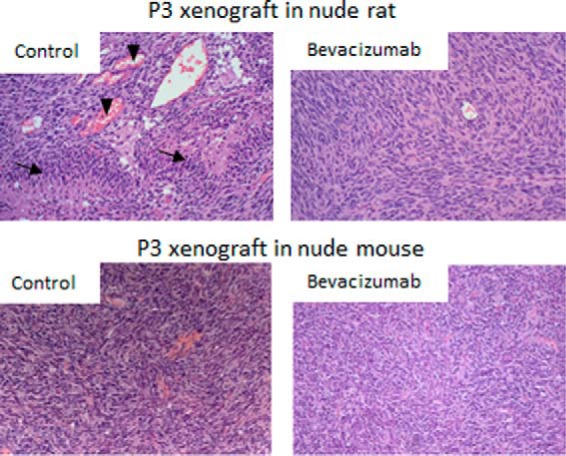
**Rat *versus* mouse model of patient-derived GBM xenograft.** Intracranial P3 GBM xenografts were generated in nude rats or nude mice as described under “Experimental Procedures.” Hematoxylin and eosin (H&E) stained sections show that GBM xenografts derived in rats display a typical strong angiogenic phenotype including visible pseudopalisading cells (arrows) and dilated blood vessels (arrowheads), whereas these features are hardly present in mice xenografts. Treatment of xenografts in rats leads to a morphological normalization of the vasculature and a strong adaptation of the tumor structure as described (4), whereas less obvious changes are observed in mice upon bevacizumab treatment. It should be noted however that a decrease in endothelial cell number can be detected in both species (4, 21).

Table I provides the list of 32 human protein candidates that were statistically differentiated after bevacizumab treatment in mouse and in rat GBM xenografts. A fold change superior to 1.0 indicates that the protein was up-regulated in bevacizumab treated samples whereas a fold change inferior to 1.0 indicates that the protein was downregulated. Interestingly, the number of differentially regulated proteins was much higher in rat than in mouse xenografts, which is most likely explained by the fact that the rat xenografts used here display a more pronounced angiogenic phenotype compared with mice. Indeed mouse xenografts harbored few pathological blood vessels and barely any necrosis compared with rat xenografts, even though they were derived from the same parental GBM (Fig. 4). As a consequence the response to bevacizumab was less dramatic in this species, as shown at the histological level on Fig. 4 and as is also reflected in the present SRM data. It should be noted that this is not because of a reduced responsiveness of mouse endothelial cells, because we have previously shown a reduction in endothelial cell number in mice in response to bevacizumab (21). Therefore we consider the proteins identified in rats as reliable biomarker candidates, even if they were not necessarily detected in the initial test material. Interestingly, the vast majority of affected proteins were downregulated after bevacizumab treatment, such as several metabolic enzymes involved in the aerobic metabolism of energy production. This included α-enolase, mitochondrial aldehyde dehydrogenase (ALDH2), fumarate hydratase, Acetyl-CoA acetyltransferase, LDHB, isocitrate dehydrogenases 1 and 2 (IDH1/2), in agreement with a reduction in mitochondrial TCA cycle activity and induction of the glycolytic pathway. We detected several proteins of the endoplasmic reticulum (ER) to be affected by the treatment. Of these, calnexin (1.28 fold) and malectin (1.31 fold) were up-regulated after bevacizumab treatment, whereas calreticulin and different isoforms of protein disulfide-isomerase (PDI A3, A4, A6) were reduced. Malectin, calnexin, and calreticulin are ER resident lectins and chaperones involved in glycoprotein production and quality control of glycoprotein secretion (30). PDI is involved in the rearrangement of disulfide bonds and functions as a chaperone to inhibit aggregation of misfolded proteins (31).

**Table I:** List of the proteins differentially expressed in GBM xenografts after bevacizumab treatment. Fold changes (FC, values represent treated over untreated) are indicated for the proteins significantly affected in mice and rats xenografts, using the Hochberg multiple comparison correction method (p-Value < 0.01)

ID	Name	Rat GBM xenografts FC	CV (%)^a^	Mice GBM xenografts FC	CV (%)^a^
Q14165	Malectin (MLEC)	1.31	12		
P27824	Calnexin (CANX)	1.28^b^	10		
P00338	l-lactate dehydrogenase A chain (LDHA)	1.16	9		
P14618	Pyruvate kinase isozymes M2 (PKM)	0.92	13		
P06733	Alpha-enolase (ENO1)	0.88	8		
P49327	Fatty acid synthase (FASN)	0.88	13		
P60174	Triosephosphatate isomerase (TPI1)	0.88	9		
P23381	Tryptophanyl-tRNA synthetase (WARS)	0.82	15		
P27797	Calreticulin (CALR)	0.77^b^	8		
P05091	Aldehyde dehydrogenase, mitochondrial (ALDH2)	0.77	13		
P23284	Peptidyl-prolyl cis-trans isomerase B (PPIB)	0.77	6		
P07237	Protein disulfide-isomerase (P4HB)	0.76^b^	10		
P13667	Protein disulfide-isomerase A4 (PDIA4)	0.76^b^	9		
P30101	Protein disulfide-isomerase A3 (PDIA3)	0.75^b^	12		
P08758	Annexin A5 (ANXA5)	0.75	9		
Q15084	Protein disulfide-isomerase A6 (PDIA6)	0.75	10		
Q9NQ88	Probable fructose-2,6-bisphosphate TIGAR (TIGAR)	0.75	12		
Q16698	2,4-dienoyl-CoA reductase, mitochondrial (DECR1)	0.74^b^	9		
Q04837	Single-stranded DNA-binding protein, mitochondrial (SSBP1)	0.73	9		
P07195	l-lactate dehydrogenase B chain (LDHB)	0.72^b^	12		
P07954	Fumarate hydratase, mitochondrial (FH)	0.72	11		
P24752	Acetyl-CoA acetyltransferase, mitochondrial (ACAT1)	0.71^b^	8		
Q06830	Peroxiredoxin 1 (PRDX1)	0.71^b^	10	0.93	10
P08670	Vimentin (VIM)	0.7	9	1.07	9
P13804	Electron transfer flavoprotein subunit alpha, mitochondrial (ETFA)	0.68^b^	10		
P30041	Peroxiredoxin 6 (PRDX6)	0.67^b^	12		
O15540	Fatty acid-binding protein, brain (FABP7)	0.64^b^	8		
P29966	Myristoylated alanine-rich C-kinase substrate (MARCKS)	0.64^b^	16	0.89	16
P30626	Sorcin (SRI)	0.63^b^	9		
O75874	Isocitrate dehydrogenase [NADP] cytoplasmic (IDH1)	0.62^b^	8	0.88^b^	8
P50440	Glycine amidinotransferase, mitochondrial (GATM)			0.8^b^	13
P48735	Isocitrate dehydrogenase [NADP], mitochondrial (IDH2)			0.92	15

^a^ Value of the heterogeneity (CV in %) of the protein estimated within untreated GBM xenografts.

^b^ Peptide transitions that are conserved (p-Value < 0.01) after Bonferroni correction (most stringent multiple comparison correction method).

**Fig. 5.**
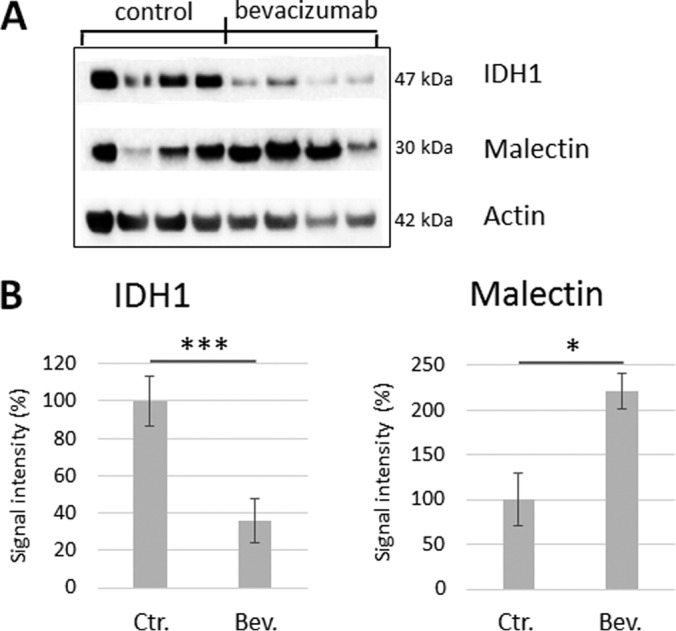
**Modulation of IDH1 and malectin in GBM xenografts after bevacizumab treatment.** (A) Western blot analysis was performed on rat GBM xenografts-treated or not with bevacizumab. In agreement with the SRM data, reduced levels of IDH1 and increased malectin were observed in four different samples. (B) Quantification of signal intensity normalized to actin. Malectin showed a 2.21 fold increase, whereas a 0.36 fold decrease was measured for IDH1. * 0.05 < *p* value < 0.1; *** *p* value < 0.001.

To confirm the SRM results, we analyzed the expression of malectin and IDH1, the most responsive proteins of our subset by Western blot analysis. Using four different GBM xenografts, we were able to confirm the decrease of IDH1 and the increase of Malectin upon bevacizumab treatment as shown in Fig. 5. The fold changes were similar to the SRM assay (malectin: 2.21 *versus* 1.31 fold by SRM, IDH1: 0.36 *versus* 0.62 fold by SRM), indicating that the SRM quantification is reliable and comparable to antibody based assays. Thus the target proteins presented here represent potential novel markers of GBM treatment response to anti-angiogenic agents.

## DISCUSSION

This is to our knowledge the first targeted proteomics study in GBM addressing the identification of biomarkers in response to anti-angiogenic treatment. Following up on our previous studies addressing the tumor escape mechanisms to bevacizumab (4, 5), we here present an efficient and optimized SRM workflow for complex cancer tissue, which allows an accurate quantification of small protein changes observed in response to treatment. In agreement with our previous work, we identified decreased levels of metabolic enzymes related to mitochondrial energy production, whereas LDHA, a key enzyme of the glycolytic pathway responsible for lactate production, was found to be induced by the treatment. We further report the up-regulation of malectin and calnexin, two lectins involved in glycoprotein maturation and quality control, which have not been previously connected to anti-angiogenic treatment. The reliability and reproducibility of the data was substantiated by Western blot analysis, which confirmed the regulation of malectin and IDH1. Thus the identified proteins represent potential novel biomarkers to assess treatment response to anti-angiogenic agents and further characterization of the candidates in clinical GBM samples is warranted.

In view of the strong heterogeneity within complex biological samples, our results highlight the importance of data normalization and assessing protein variability for SRM analysis. We show how protein variability between samples can be assessed and taken into account in comparative studies. In the context of GBM, previous proteomic studies were focused on the analysis of human plasma (32), serum (33) or biopsies (34, 35) and in most cases human GBM cell lines (36–39). A recent study monitored 65 targeted proteins by SRM in the secretome of GBM cell lines expressing different variants of the epidermal growth factor receptor (EGFR) (40). However, no study has so far addressed a therapeutic response, which is likely to induce only minor changes in protein expression levels. We report small but statistically significant fold changes between 0.62–1.31-fold, which are only measurable with a highly sensitive and accurate approach. This makes SRM the method of choice to monitor small protein concentration changes in complex biological samples.

Interestingly the induction of LDHA was not only seen in tumor cells but also in the non-neoplastic host compartment, an observation that was possible because of the species specific SRM workflow established here. By applying the workflow to human GBM developed in a rodent background, emphasis was put on the discrimination of different proteome species within the same sample. Thus an automated workflow for species specific peptide selection and the precise quantification of protein content in human tumor *versus* non-neoplastic host cells has been developed. Our in house developed software *PeptideManager,* released in open-access, provided an efficient and fast solution in this crucial selection step (27). We have previously applied iTRAQ analysis in GBM xenograft samples, where the species specificity was established a posteriori in the data analysis step (24, 41). Using SRM, the species-specific peptide selection allows an upfront determination of human and rodent proteins within the same sample.

The observation that several enzymes involved in aerobic metabolism were reduced upon bevacizumab treatment is in agreement with our previous studies demonstrating an induction of tumor hypoxia and increased glucose consumption through the glycolytic pathway. This metabolic switch is reflected here by reduced levels of α-enolase, aldehyde dehydrogenase (ALDH2), fructose 1–6 bisphosphate TIGAR, fumarate hydratase, acetyl-CoA acetyltransferase, IDH1, and IDH2. Furthermore, although LDHA was increased, the LDHB subunit responsible for lactate to pyruvate conversion (42), was reduced, further supporting reduced oxidative phosphorylation in bevacizumab treated tumors. Interestingly we observed a slight reduction in pyruvate kinase M2 (PKM2; 0.92 fold), the PK isoform that is often associated with tumor specific expression and aerobic glycolysis (43). The role of PKM2 in hypoxic glycolysis is however less clear and recent data suggests that the regulation of PKM1 and PKM2 isoforms in cancer may be more complicated than originally thought (44–46).

Among other proteins which were modulated after treatment we observed several ER resident proteins, including the most strongly increased proteins malectin and calnexin, whereas calreticulin and PDIs were reduced. Malectin is a membrane-anchored carbohydrate-binding protein in the ER and has been proposed to be involved in the early steps of protein N-glycosylation (47). Highly conserved in animals, malectin specifically binds diglucosylated glycans, whereas calnexin and calreticulin bind to monoglucosylated glycan side chains. These lectin molecular chaperones play an important role in assisting the maturation and quality control of glycoproteins in the early secretory pathway (30). It was recently shown that Malectin is induced by ER stress and contributes to reduced secretion of defective proteins when the ER quality control machinery is poorly functioning (48). Whether the modulation of malectin, calnexin, calreticulin, and PDIs is a result of hypoxia induced ER stress in response to bevacizumab remains to be determined. It is interesting to speculate that glycoprotein production and maturation may be affected by anti-angiogenic treatment, suggesting that glycoproteins could serve as important response markers.

A recent study reported that high baseline plasma levels of matrix metalloproteinase 2 (MMP2) are associated with prolonged tumor control and patient survival after bevacizumab treatment (49). MMP2 was not present in our protein list, however it will be interesting to establish MMP2 levels by SRM and determine its response in our GBM xenografts. In particular this would allow to determine the origin of MMP2 from tumor or stromal tissue. In future studies, the current list of SRM target proteins could be expanded to include potential markers related to other anti-angiogenic agents (*e.g.* cediranib, celingitide). These include *e.g.* basic fibroblast growth factor (bFGF), angiopoietin-2, stromal derived factor-1 (SDF1), but also markers of macrophages and circulating endothelial cells (50, 51). The establishment of a panel of target proteins modulated upon anti-angiogenic therapy may provide the robustness required for biomarker based patient stratification. Indeed the identification of molecular biomarkers to predict response and to signal resistance in GBM is a high clinical priority and ongoing clinical studies with anti-angiogenic agents aim to achieve appropriate patient enrichment and effective combination therapies with complementary approaches (52).

In conclusion, we provide an accurate and highly sensitive SRM workflow for establishing tumor specific protein differences in complex biological samples, and determine novel protein candidates that are modulated in response to anti-angiogenic treatment in GBM. The significance of these in clinical GBM samples and as potential markers of efficacy should be further explored.

## Supplementary Material

Supplemental Data

## Footnotes

^1^ The abbreviations used are:

GBM: Glioblastoma
IDH: Isocitrate dehydrogenase
MRM: Multiple Reaction Monitoring
SIL: Stable Isotope Labeled
SRM: Selected Reaction Monitoring
VEGF: Vascular Endothelial Growth Factor
MRI: magnetic resonance imaging
TCA: tricarboxylic acid cycle
ER: endoplasmic reticulum.
