# Supplementary material for: Targeted Proteomics to Assess the Response to Anti-Angiogenic Treatment in Human Glioblastoma (GBM)
Source: Mol Cell Proteomics. 2015 Aug 4;15(2):481–92. doi: 10.1074/mcp.M115.052423 (PMC4739668; doi:10.1074/mcp.M115.052423)
Supplement: Supplemental Data [file supp_15_2_481__index.html]

Targeted Proteomics to Assess the Response to Anti-Angiogenic Treatment in Human Glioblastoma — Targeted Proteomics to Assess the Response to Anti-Angiogenic Treatment in Human Glioblastoma (GBM) — Targeted Proteomics in Bevacizumab Treated GBM — Supplemental Data 

# Targeted Proteomics to Assess the Response to Anti-Angiogenic Treatment in Human Glioblastoma (GBM)

## Supplemental Data

- Supplemental Table 1 - protein list
- Supplemental Table 2 - transition list
- Supplemental Table 3 - normalization factors
- Supplemental Table 4 - variability assessment
- Supplemental Figure 1 - Heterogeneity assessment of the proteins of interest within GBM xenografts.
- Supplemental data - PEPTIDEATLAS DATASET Supplemental legends
