# Supplementary material for: Targeted Proteomics to Assess the Response to Anti-Angiogenic Treatment in Human Glioblastoma (GBM)
Source: Mol Cell Proteomics. 2015 Aug 4;15(2):481–92. doi: 10.1074/mcp.M115.052423 (PMC4739668; doi:10.1074/mcp.M115.052423)
Supplement: Supplemental Data [file supp_M115.052423_mcp.M115.052423-5.pdf]

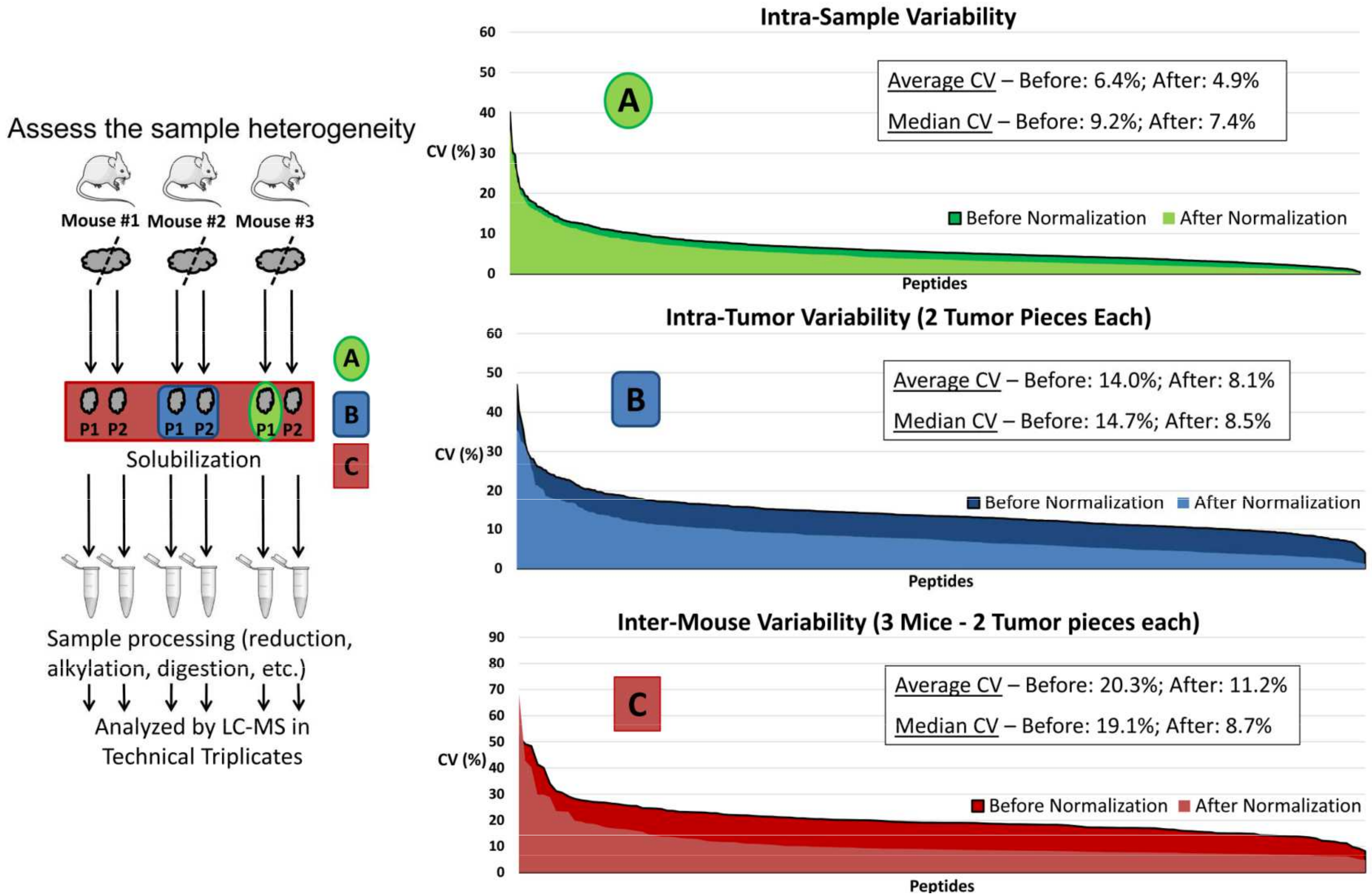

<u>Supplemental Figure 1.</u> Heterogeneity assessment of the proteins of interest within GBM xenografts. The CVs of each peptide area (sum of the areas of its transitions) for every endogenous peptide within each tumor piece (intra-sample variability) for all the samples are indicated in decreasing order in the diagram A before and after normalization (828 peptide/measures in total). The CVs of each transition for every endogenous peptide within the two tumor pieces (intra-tumor variability) of each mouse are indicated in decreasing order in the diagram B before and after normalization (414 peptides/measures in total). The CV of each transition for every endogenous peptide within all the mice (all the tumor pieces) (inter-mouse variability) are indicated in decreasing order in the diagram C before and after normalization (138 peptides/measures in total). The values of the average and median CVs before and after normalization are indicated within each diagram. The effect of the normalization is minor at the intra-sample level (diagram A) but far more significant at the intra-tumor and inter-mouse levels (inter-sample levels: diagram B and C).
