# Supplementary material for: Targeted Proteomics to Assess the Response to Anti-Angiogenic Treatment in Human Glioblastoma (GBM)
Source: Mol Cell Proteomics. 2015 Aug 4;15(2):481–92. doi: 10.1074/mcp.M115.052423 (PMC4739668; doi:10.1074/mcp.M115.052423)
Supplement: Supplemental Data [file supp_M115.052423_mcp.M115.052423-6.pdf]

1   **PEPTIDEATLAS DATASET**

2   **PeptideAtlas Dataset Heterogeneity.** SRM dataset of the heterogeneity assessment of the protein
3   candidates within GBM mouse xenografts.

4   Identifier: PASS00671

5   Username: PASS00671

6   Password: HY8855xf

7   Full URL: <ftp://PASS00671:HY8855xf@ftp.peptideatlas.org>

8   **PeptideAtlas Dataset Mice – bevacizumab treated or not.** SRM dataset of the comparative study of
9   the protein candidates within GBM mouse xenografts treated or not with bevacizumab.

10   Identifier: PASS00680

11   Username: PASS00680

12   Password: JC9947c

13   Full URL: <ftp://PASS00680:JC9947c@ftp.peptideatlas.org>

14   **PeptideAtlas Dataset Rat – bevacizumab treated or not.** SRM dataset of the comparative study of the
15   protein candidates within GBM rat xenografts treated or not with bevacizumab.

16   Identifier: PASS00681

17   Username: PASS00681

18   Password: XL4399d

19   Full URL: <ftp://PASS00681:XL4399d@ftp.peptideatlas.org>

20

SUPPLEMENTAL DATA

Supplemental Table 1. List of screened proteins and peptides, and the corresponding SIL peptides ordered.

Supplemental Table 2. List of the transitions monitored by the designed SRM assay.

Supplemental Table 3. Estimation of normalization factors with house-keeping proteins.
Normalization factors estimated for the normalization procedure with the human house-keeping proteins. The coefficients of variation (CVs in %) indicate the variability of the correction value amongst the different transitions monitored for the peptides, surrogates of the human house-keeping proteins.

Supplemental Table 4. Variability of the protein candidates within GBM mouse xenografts.

Supplemental Figure 1. Heterogeneity assessment of the proteins of interest within GBM xenografts. The CVs of each peptide area (sum of the areas of its transitions) for every endogenous peptide within each tumor piece (intra-sample variability) for all the samples are indicated in decreasing order in the diagram A before and after normalization (828 peptide/measures in total). The CVs of each transition for every endogenous peptide within the two tumor pieces (intra-tumor variability) of each mouse are indicated in decreasing order in the diagram B before and after normalization (414 peptides/measures in total). The CV of each transition for every endogenous peptide within all the mice (all the tumor pieces) (inter-mouse variability) are indicated in decreasing order in the diagram C before and after normalization (138 peptides/measures in total). The values of the average and median CVs before and after normalization are indicated within each diagram. The effect of the normalization is minor at the intra-sample level (diagram A) but far more significant at the intra-tumor and inter-mouse levels (inter-sample levels: diagram B and C).
